# Clades of Photosynthetic Bacteria Belonging to the Genus *Rhodopseudomonas* Show Marked Diversity in Light-Harvesting Antenna Complex Gene Composition and Expression

**DOI:** 10.1128/mSystems.00006-15

**Published:** 2015-12-22

**Authors:** Kathryn R. Fixen, Yasuhiro Oda, Caroline S. Harwood

**Affiliations:** Department of Microbiology, University of Washington, Seattle, Washington, USA; University of California, Davis

**Keywords:** transcriptomics, biotechnology, phototrophs

## Abstract

Many photosynthetic bacteria have peripheral light-harvesting (LH) antenna complexes that increase the efficiency of light energy capture. The purple nonsulfur photosynthetic bacterium *Rhodopseudomonas palustris* produces different types of LH complexes under high light intensities (LH2 complex) and low light intensities (LH3 and LH4 complexes). There are multiple *pucBA* operons that encode the α and β peptides that make up these complexes. However, low-resolution structures, amino acid similarities between the complexes, and a lack of transcription analysis have made it difficult to determine the contributions of different *pucBA* operons to the composition and function of different LH complexes. It was also unclear how much diversity of LH complexes exists in *R. palustris* and affiliated strains. To address this, we undertook an integrative genomics approach using 20 sequenced strains. Gene content analysis revealed that even closely related strains have differences in their *pucBA* gene content. Transcriptome analyses of the strains grown under high light and low light revealed that the patterns of expression of the *pucBA* operons varied among strains grown under the same conditions. We also found that one set of LH2 complex proteins compensated for the lack of an LH4 complex under low light intensities but not under extremely low light intensities, indicating that there is functional redundancy between some of the LH complexes under certain light intensities. The variation observed in LH gene composition and expression in *Rhodopseudomonas* strains likely reflects how they have evolved to adapt to light conditions in specific soil and water microenvironments.

**IMPORTANCE** *Rhodopseudomonas palustris* is a phototrophic purple nonsulfur bacterium that adapts its photosystem to allow growth at a range of light intensities. It does this by adjusting the amount and composition of peripheral light-harvesting (LH) antenna complexes that it synthesizes. *Rhodopseudomonas* strains are notable for containing numerous sets of light-harvesting genes. We determined the diversity of LH complexes and their transcript levels during growth under high and low light intensities in 20 sequenced genomes of strains related to the species *Rhodopseudomonas palustris*. The data obtained are a resource for investigators with interests as wide-ranging as the biophysics of photosynthesis, the ecology of phototrophic bacteria, and the use of photosynthetic bacteria for biotechnology applications.

## Abstract (precis)

*Rhodopseudomonas palustris* is a phototrophic purple nonsulfur bacterium that adapts its photosystem to allow growth at a range of light intensities. It does this by adjusting the amount and composition of peripheral light-harvesting (LH) antenna complexes that it synthesizes. *Rhodopseudomonas* strains are notable for containing numerous sets of light-harvesting genes. We determined the diversity of LH complexes and their transcript levels during growth under high and low light intensities in 20 sequenced genomes of strains related to the species *Rhodopseudomonas palustris*. The data obtained are a resource for investigators with interests as wide-ranging as the biophysics of photosynthesis, the ecology of phototrophic bacteria, and the use of photosynthetic bacteria for biotechnology applications.

## INTRODUCTION

Purple nonsulfur bacteria (PNSB) have served as models for studies of photosynthesis because they have much simpler systems of light energy conversion than do green plants or cyanobacteria. PNSB generate ATP by cyclic photophosphorylation in which electrons that have been energized by light leave a bacteriochlorophyll (Bchl)-containing reaction center, are cycled through a proton-pumping electron transport chain, and are then returned to the reaction center. This cyclic flow of electrons does not generate oxygen, and the process is not obligatorily linked to carbon dioxide fixation. Light-harvesting (LH) antenna complexes act in conjunction with reaction centers to increase the efficiency of light energy capture by PNSB.

Much of our knowledge about the structure and energy transfer events of LH complexes in PNSB has been obtained from work carried out with the species *Rhodopseudomonas palustris*. Most PNSB, including *R. palustris*, have peripheral LH complexes known as LH2 that are involved in capturing light and transferring that energy to the LH1 complex, which surrounds the reaction center to form a core photoconversion complex. Both types of LH complexes are circular oligomers of a protomer composed of hydrophobic polypeptides α and β that interact with the light-absorbing pigments bacteriochlorophyll (Bchl) and carotenoids (Car). The LH1 complex from *R. palustris* contains 15 pairs of the α and β peptides that bind one Car and two molecules of Bchl*a*, which are tightly coupled and absorb light maximally at a wavelength of 875 nm (1). The LH2 complex of *R. palustris* is a nonameric ring of α/β pairs that each bind one Car, one monomeric Bchl*a* molecule that absorbs light at a wavelength of 800 nm (B800), and two more tightly coupled Bchl*a* molecules that absorb light at 850 nm (B850) (2).

Genomes of strains related to *R. palustris* contain more genes encoding LH2-like complexes than any other PNSB sequenced to date, with one strain, BisA53, containing seven sets of LH2-like genes (3). These multiple gene sets allow *Rhodopseudomonas* strains to synthesize alternative LH2-like complexes called LH2′, LH3, and LH4 complexes. *R. palustris* adjusts the amount and composition of its LH complexes in response to changes in environmental conditions (4–8). This is most apparent when the photosynthetic membranes of cells grown under high light (HL) intensities (30 µmol photons/m^2^/s) are compared to those of cells grown under low light (LL) intensities (4 µmol photons/m^2^/s). Under HL, LH2 is the predominant peripheral LH complex in *Rhodopseudomonas* membranes, although BisA53 also makes an additional LH2 complex known as LH2′ that absorbs light at wavelengths of 800 nm and 840 nm (9). Under LL, some *Rhodopseudomonas* strains produce LH4, which maximally absorbs light at 800 nm (5, 9–11). BisA53 also makes an LH3 complex that absorbs 800-nm and 820-nm light (3, 9). The α and β peptides of different complexes have been shown to form a heterogeneous complex so that each LH complex ring contains a mixture of different α/β protein pairs under LL in *R. palustris*, and this is thought to prevent exciton trapping and backward energy transfer from LH1 for more efficient light harvesting (12, 13).

Since they contain so many sets of light-harvesting genes, *Rhodopseudomonas* strains provide a unique system to understand the extent to which a photosynthetic bacterium has evolved to adjust to fluctuations in light intensity. To address this opportunity, we undertook an integrative genomics approach using 16 sequenced *Rhodopseudomonas* strains that have ≥99% 16S rRNA sequence identity and greater than 97% average nucleotide identity (ANI) with *R. palustris* CGA009 (14) and thus represent closely related strains of a distinct clade of *Rhodopseudomonas* (Table 1; see also Table S1 in the supplemental material). It should be noted that these strains have an average ANI of 93.2% compared to strain ATCC 17001, the type strain of *R. palustris* (see Table S1). Four additional *Rhodopseudomonas* strains were also included in this study. One of the strains, DX-1, has an ANI of 90% compared to either strain CGA009 or strain ATCC 17001 and therefore forms its own clade. The genomes of the remaining three strains, BisB5, BisB18, and BisA53, have been previously described (3). These three strains share <98% identity in 16S rRNA sequence to *R. palustris* CGA009 and are quite different from each other. Here, we collectively refer to this cohort of 20 strains as members of the genus *Rhodopseudomonas*. We identified and compared the complements of light-harvesting genes in each genome and analyzed the transcriptomes of the 20 *Rhodopseudomonas* strains grown under HL and LL. We determined that even closely related *Rhodopseudomonas* strains have differences in LH gene content and expression, even under the same growth conditions. We also found that the LH2 complex could compensate for the lack of an LH4 complex under LL intensities but not under extremely LL (ELL) intensities. Taken together, these data likely reflect how *Rhodopseudomonas* strains have diversified to occupy specific microenvironments in terrestrial soil and water environments that have different levels of exposure to light.

**TABLE 1:** LH gene composition in closely related *Rhodopseudomonas* strains

*Rhodopseudomonas* strain	% 16S rRNA identity to CGA009	No. of *pucB* genes	No. of *pucA* genes encoding:					
			LH2A	LH2B	LH2E	LH3	LH4	LH2′
TIE-1	100	5	1	1	1	1	1	0
1a1	100	5	1	1	1	1	1	0
AP1	100	5	1	1	1	1	1	0
BIS3	100	5	1	1	1	1	1	0
DCP3	99.9	5	1	1	1	1	1	0
DSM126	100	5	1	1	1	1	1	0
KD1	99.9	5	1	1	1	1	1	0
NCIB8288	99.8	5	1	1	1	1	1	0
RCH350	99.9	5	1	1	1	1	1	0
RCH500	100	5	1	1	1	1	1	0
RSP24	100	5	1	1	1	1	1	0
CGA009	100	5	1	1	1	Pseudo^a^	1	0
0001L	100	5	1	1	1	Pseudo	1	0
ATCC 17007	100	5	1	1	1	Pseudo	1	0
CEA001	100	5	1	1	1	Pseudo	1	0
DX-1	99.4	3	1	1	1	0	0	0
DSM8283	100	3	1	1	1	0	0	0
BisB5	97.5	5	1	1	1	0	2	0
BisB18	97.3	4	1	1	0	2	0	0
BisA53	97.8	7	0	1	4	2	0	1

^a^ Pseudo, classified as a pseudogene.

10.1128/mSystems.00006-15.1 Table S1  Relative transcript levels for all LH genes and growth rates of 20 *Rhodopseudomonas* strains grown under high light intensity (30 µmol photons/m^2^/s) and low light intensity (4 µmol photons/m^2^/s) under nitrogen-fixing conditions. Download Table S1, XLSX file, 63 KB. Copyright © 2015 Fixen et al. 2015 Fixen et al. This content is distributed under the terms of the Creative Commons Attribution 4.0 International license.

## RESULTS

### Inventory of genes encoding peripheral LH peptides in 20 *Rhodopseudomonas* strains.

By convention, LH genes are designated *pucBA*, with *pucA* encoding the α peptide and *pucB* encoding the β peptide of a light-harvesting complex monomer. The α peptides of the LH2-like complexes are 60 to 65 amino acids in length and can be distinguished based on the identities of two or three amino acids, which result in differences in Bchl binding and thus their light absorption properties. The β peptides are approximately 50 amino acids in length but are almost identical in their amino acid sequences, with greater than 90% identity. Thus, we used the amino acid sequences of the predicted α peptides to determine which types of LH complexes are encoded in each genome. As shown in Table 2, four different types of LH peptides, LH2, LH2′, LH3, and LH4, can be distinguished based on amino acid signatures (2, 9, 11, 15, 16). *Rhodopseudomonas* strains have multiple sets of LH2 genes, which we categorized as LH2A, LH2B, and LH2E based on their amino acid identities to LH2 genes in our reference strain CGA009 (see Table S1 in the supplemental material).

**TABLE 2:** Light-harvesting complexes produced by *Rhodopseudomonas*^a^

Light-harvesting antenna complex	Light absorption peak(s) (nm)	Amino acid(s) in the α peptide used for identification
LH2	800 and 850 nm	Y_44_, W_45_
LH2′	800 and 840 nm	Y_44_, M_45_
LH3	800 and 820 nm	F_44_, M_45_, or F_45_
LH4	800 nm	M_26_, F_44_, M_45_

^a^ Classification of LH complexes is taken from reference 9.

A compilation of the number and types of LH peptides encoded in the genomes of the 20 *Rhodopseudomonas* strains is shown in Table 1. Our reference strain CGA009 has three operons encoding LH2 complexes (*pucBAa*, *pucBAb*, and *pucBAe*), one operon that encodes the LH4 complex (*pucBAd*), and one operon, *pucBAc*, which would encode an LH3 complex except that *pucAc* is a pseudogene due to a 2-bp insertion in the coding sequence. All locus numbers for each of the *pucBA* homologs from each strain are listed in Table S1 in the supplemental material. Of the *Rhodopseudomonas* strains that are most closely related to CGA009 (≥99.5% identity in 16S rRNA sequence), 11 strains had all three operons encoding LH2, one operon encoding LH4, and an intact operon encoding an LH3 complex, and this likely reflects the most prevalent combination of *pucBA* genes contained in *Rhodopseudomonas* strains closely related to CGA009 (Table 1). Three strains, 0001L, ATCC 17007, and CEA001, had exactly the same *pucBA* gene composition as CGA009, including the same 2-bp insertion in the coding sequencing of *pucAc* that renders it a pseudogene (Table 1). This suggests that this mutation is not simply a result of laboratory domestication but may have been passed from a shared ancestor of these four strains. Surprisingly, the genomes of two strains, DX-1 and DSM8283, had three operons encoding LH2 complexes but did not encode either an LH3 or LH4 complex (Table 1). These two strains are likely adapted to environments with higher light intensities and either did not undergo the same gene duplication event resulting in LH3/4 or lost the genes encoding LH3/4.

Table 1 also shows that *Rhodopseudomonas* strains that are not as closely related to CGA009 (<98% identity in 16S rRNA sequence) have a greater diversity in the number and type of LH peptides that they encode. BisA53 stands out as having the most LH complexes, and unlike the other strains, only 7 of its 8 *pucA* genes are found in tandem with a *pucB* gene (see Table S1 in the supplemental material).

### Evolutionary relationships among the peripheral LH complexes of 20 *Rhodopseudomonas* strains.

**FIG 1:**
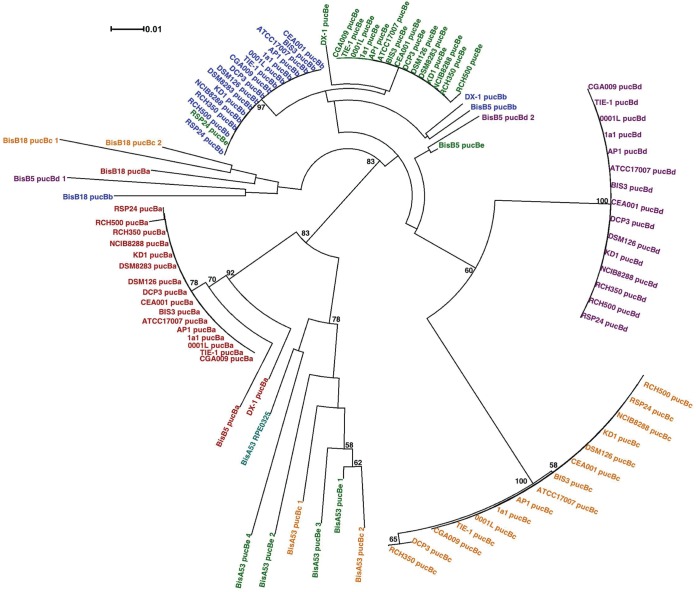
Evolutionary relationships among the β peptides of LH complexes from 20 *Rhodopseudomonas* strains. The phylogenetic tree of *pucB*, encoding the β peptide of LH complexes, is shown. Sequences are identified by the name of the strain from which they originated and color coded based on which LH β peptide complex they encode. The scale indicates the number of base substitutions per site. Bootstrap values are represented as percentages of 10,000 replicates, and only nodes with values of 50% or greater are shown. Details of phylogenetic analysis are discussed in Materials and Methods.

**FIG 2:**
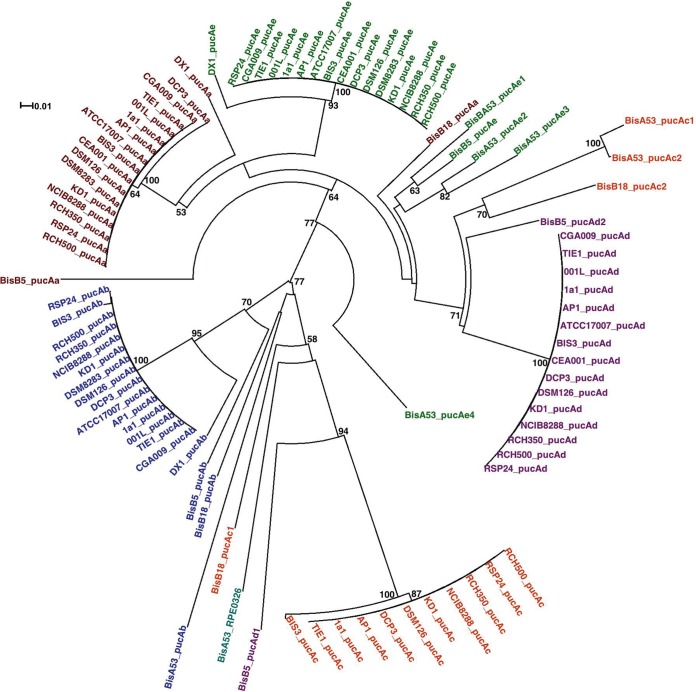
Evolutionary relationships among the α peptides of LH complexes from 20 *Rhodopseudomonas* strains. The phylogenetic tree of *pucA*, encoding the α peptide of LH complexes, is shown. Sequences are identified by the name of the strain from which they originated and color coded based on which LH α peptide complex they encode. The scale indicates the number of base substitutions per site. Bootstrap values are represented as percentages of 10,000 replicates, and only nodes with values of 50% or greater are shown. Details of phylogenetic analysis are discussed in Materials and Methods.

To gain insight into the evolutionary relationships among the LH complexes of different strains, we constructed phylogenetic trees from the *pucB* and *pucA* nucleotide sequences (Fig. 1 and 2). A study looking at the evolution of LH complexes in PNSB proposed that the phylogenetic tree of the β peptide most accurately reflects the genuine evolutionary relationships between the LH genes because the β peptides have a longer conserved sequence than the α peptides and the β tree closely matched the 16S rRNA gene tree (17). In agreement with this, the *pucB* tree for the 20 *Rhodopseudomonas* strains shows that the strains with almost 100% identity in their 16S rRNA sequence cluster together and strains with more divergent 16S rRNA sequences (DX-1, BisB5, BisB18, and BisA53) have more divergent *pucB* sequences (Fig. 1). In particular, the *pucB* sequences of BisA53 and BisB18 form two distinct clusters. This suggests that the LH genes from these strains are the result of gene duplications that occurred after BisB18 and BisA53 diverged from the other *Rhodopseudomonas* strains. This is in contrast to the αtree, where the BisB18 and BisA53 sequences are intermingled with the other strains and do not form distinct clusters (Fig. 2). From the *pucB* tree, there are two separate lineages of the LH genes, the *pucBa* lineage and the *pucBbcde* lineage. It appears that the BisB18 LH genes arose from a progenitor of the *pucBbcde* lineage and the BisA53 LH genes arose from a progenitor of the *pucBa* lineage.

The *pucB* tree also suggests that *pucBbcde* arose from a gene duplication or gene transfer event (Fig. 1). There are multiple lines of evidence that support this. The *pucBb* and *pucBe* from CGA009 differ by only four nucleotides and are identical in their amino acid sequences. Also, the promoters for *pucBb*, *pucBe*, and *pucBd* show significant similarity in their regulatory elements (18). Finally, *pucBAe* and *pucBAd* have similar flanking genes in some *Rhodopseudomonas* strains, which suggests that a duplication or gene transfer event occurred (9).

As observed in reference 9, the *pucA* tree is divided into two distinct clades (Fig. 2). In one clade, *pucAb* and *pucAc* sequences, which encode α peptides with a polyproline/polyalanine C-terminal extension, cluster together, and *pucA* sequences that do not have the C-terminal extension cluster in the other clade. In *Rubrivivax gelatinosus*, the C-terminal extension appears to play a role in assembly of the LH2 complex (19).

### Transcription profiles of peripheral LH genes in 20 *Rhodopseudomonas* strains under HL and LL.

Three different proteomics studies looking at LH peptide composition under HL and LL have each reported different findings (10, 20, 21). These differences could be due to differences in the *Rhodopseudomonas* strain or growth conditions used in the study. To address these issues, we used transcriptome sequencing (RNA-seq) to measure the transcript levels of the LH genes in the 20 *Rhodopseudomonas* strains grown under the same HL and LL conditions. The LH genes are the most highly expressed genes in the genomes of each of the strains under both HL and LL conditions. While the majority of genes in cells grown under HL and LL have relative transcript levels of between 50 and 250 rpkm (reads per kilobase per million uniquely mapped reads), the LH genes can have relative transcript levels upwards of 200,000 (Table 3). It is unclear if these high transcript levels are due to high levels of transcription *per se* or to a high level of stability of the mRNA transcripts, but certainly the LH proteins are very abundant in cells.

**TABLE 3:** Comparison of relative transcript levels of LH genes to those of other photosynthesis and housekeeping genes in *R. palustris* CGA009

Gene (locus no.)^a^	Product	Relative transcript levels (mean rpkm^b^)	
		Low light	High light
*pucBc* (RPA3009)	LH3 beta chain C	279,889	5,819
*pucBa* (RPA2654)	LH2 beta chain A	215,767	346,456
*pufB* (RPA1525)	LH1 beta chain	203,410	129,965
*pucBd* (RPA3013)	LH4 beta chain D	156,693	2,996
*pufA* (RPA1526)	LH1 alpha chain	85,516	49,070
*pucAd* (RPA3012)	LH4 alpha chain D	32,046	656
*pucAa* (RPA2653)	LH2 alpha chain A	24,466	38,019
*pufM* (RPA1528)	Reaction center M protein	4,158	1,962
*pucAe* (RPA1492)	LH2 alpha chain E	2,332	4,125
*pucAb* (RPA4292)	LH2 alpha chain B	2,207	2,891
*pucBe* (RPA1491)	LH2 beta chain E	1,662	2,445
*pucBb* (RPA4291)	LH2 beta chain B	1,033	1,389
*dnaA* (RPA0001)	Chromosome replication initiation protein	290	307
*rpoD* (RPA1288)	RNA polymerase sigma 70 subunit	277	310

^a^ All locus numbers refer to the *R. palustris* CGA009 locus number.

^b^ rpkm, reads per kilobase per million uniquely mapped reads.

**FIG 3:**
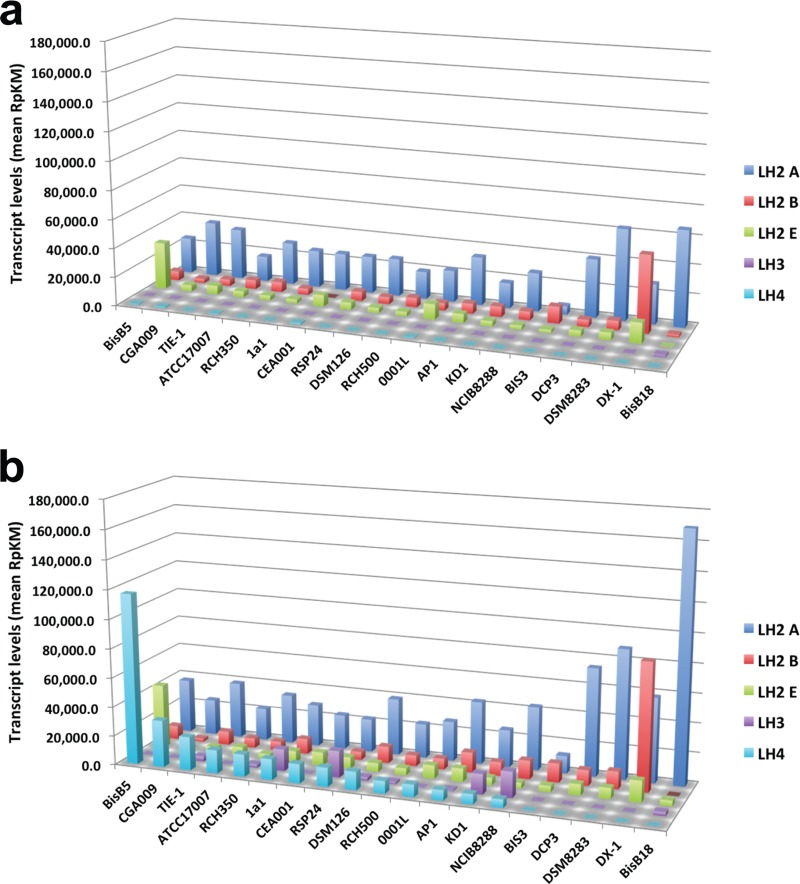
Transcript levels of LH genes in *Rhodopseudomonas* strains under HL and LL intensity. The relative transcript levels measured as reads per kilobase per million uniquely mapped reads (rpkm) are shown for all *pucA* genes in each strain grown under HL intensity (a) or LL intensity (b). All *pucA* genes were classified as LH2A, LH2B, LH2E, LH3, or LH4 based on the type of LH complex that they encode. BisA53 was not included because its genes encode a large number of LH complexes, including a unique LH complex not found in the other strains. The transcript levels for all BisA53 *pucA* genes can be found in Table S1 in the supplemental material.

Since the α peptide determines the type of LH complex produced, we focused on analyzing the expression levels of the *pucA* genes in each of the 20 *Rhodopseudomonas* strains. Transcript levels of the *pucB* gene under HL and LL can be found in Table S1 in the supplemental material. We observed marked strain-to-strain variations in the expression levels of *pucA* genes (Fig. 3), but several trends are apparent. As expected, very little expression of the *pucA* genes encoding the α peptide of the LH3 and LH4 complexes was observed in cells grown in high light (Fig. 3a). Of the three LH2 genes, the genes encoding the LH2A complex tended to be the most highly expressed LH2-encoding genes under both HL and LL (Fig. 3), suggesting that the LH2A complex may predominate in photosynthetic membranes. BisB5 and DX-1, two strains that are among the most divergent of the strains that we tested, showed much higher levels of expression of the genes encoding the LH2E and LH2B complexes compared to the LH2A complex.

**FIG 4:**
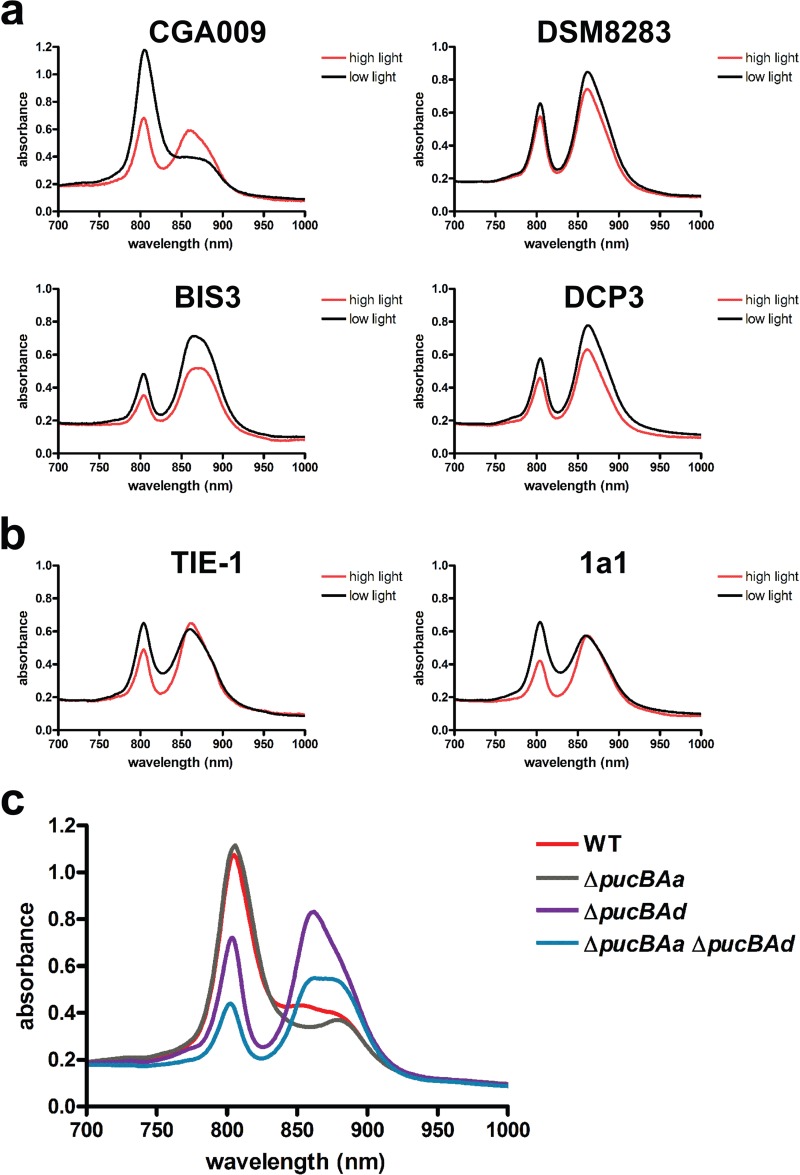
Absorbance spectra of intact *Rhodopseudomonas* cells to detect LH complexes. Absorption spectra of intact cells allow detection of LH2 (absorbance, ~800 and 860 nm), LH3 (absorbance, ~800 and 820 nm), and LH4 (absorbance, ~800 nm) complexes. (a) Absorption spectra of intact *R. palustris* CGA009 and *Rhodopseudomonas* strains that do not encode an LH3 or LH4 complex (DSM8283) or do not express genes encoding LH3 or LH4 (BIS3 and DCP3) under LL. (b) Absorption spectra of *Rhodopseudomonas* strains that encode and express the LH3 complex. (c) Absorption spectra of wild-type *R. palustris* CGA009 or *R. palustris* CGA009 with in-frame deletions of *pucBAa* (Δ*pucBAa*), *pucBAd* (Δ*pucBAd*), or both *pucBAa* and *pucBAd* (Δ*pucBAa* Δ*pucBAd*) grown under LL.

Previous studies with BisA53 and CGA009 have shown that LH3 and LH4 complexes are synthesized under LL intensity. Consistent with this, we saw an increase in transcription of *pucAd*, which encodes the α peptide of LH4, in most strains (Fig. 3b). Exceptions were DX-1 and DSM8283, which do not encode LH4, and BisB18, which has a dysfunctional LH4 regulatory pathway due to an incomplete *bphP2* regulatory gene. Surprisingly, BIS3 and DCP3 encode an LH4 but exhibited almost no expression of this operon under LL intensity (Fig. 3b; see Table S1 in the supplemental material). To verify that these strains did not synthesize the LH4 complex, the absorption spectrum of intact cells for each of these strains was determined. As shown in Fig. 4a, the absorption spectra of BIS3 and DCP3 did not show synthesis of the LH4 complex, and their spectra looked similar to that of DSM8283, which does not encode the LH4 operon. Both strains appear to have an intact set of LH4 regulatory genes.

A number of strains also showed an increase in expression of *pucAc*, which encodes the α peptide of LH3 when grown in LL (see Table S1 in the supplemental material). To determine if these strains exhibit absorption at 800 nm and 820 nm, which is characteristic of the LH3 complex, the absorption spectra of two strains, TIE-1 and 1a1, were determined. As shown in Fig. 4b, there was no clear peak of absorption at 820 nm. It is possible that the absorption peak at 800 nm hides the peak at 820 nm. It is also possible that the classification used to determine operons that encode an LH3 complex does not hold true in all cases.

### Redundancy between the LH complexes.

From energy transfer studies, it is clear that the LH4 complex is important for efficient light harvesting under LL intensities (12). From this work, we expected that there would be a correlation between the growth rate of these strains and LH4 gene expression under LL intensity. Surprisingly, there was no correlation between doubling time and LH4 gene expression under LL intensity (see Table S1 in the supplemental material). One possibility was that the other LH complexes compensate for a lack of LH4 synthesis. Consistent with this, strains that had no LH4 gene expression tended to have increased expression of the LH2A genes (Fig. 3b). To test this possibility, we constructed in-frame deletions in the LH2A operon (Δ*pucBAa*), the LH4 operon (Δ*pucBAd*), or in both operons (Δ*pucBAa* Δ*pucBAd*) in strain CGA009 and measured the growth rates of the mutants under HL and LL. To verify that these deletions affect LH complex synthesis under LL, the absorption spectra of intact cells were determined. As expected when grown under LL, the Δ*pucBAd* strain was unable to produce any LH4 (Fig. 4c). Cells that were unable to produce the LH2A complex (Δ*pucBAa*) showed a slight decrease in absorbance at 860 nm when grown under LL, and cells that were unable to produce either the LH2A or LH4 complex (Δ*pucBAa* Δ*pucBAd*) showed the greatest decrease in absorption and produced the smallest amount of LH complex under LL (Fig. 4c). As shown in Table 4, the LH4 mutant grew at about the same rate as the wild-type parent. However, the double mutant that had deletions in both the LH2A and LH4 genes grew more slowly under LL conditions. This indicates that at low light, the LH2A complex can compensate for a lack of LH4. When we dropped to extremely LL (ELL) intensities (<1 µmol photons/m^2^/s), the LH4 mutant grew more slowly than both the wild type and the LH2A mutant (Table 4), indicating that the LH4 complex is required under ELL conditions.

**TABLE 4:** Effect of light intensity on growth of *R. palustris* CGA009 wild type and *pucBA* mutants

Genotype/phenotype	Doubling time (h)^a^		
	High light (30 µmol photons/m^2^/s)	Low light (4 µmol photons/m^2^/s)	Extremely low light (<1 µmol photons/m^2^/s)
Wild type	10 (0.4)	20 (3)	185 (4)
Δ*pucBAa/*ΔLH2A	10 (0.5)	20 (2)	195 (3)
Δ*pucBAd/*ΔLH4	10 (0.3)	22 (2)	275 (26)
Δ*pucBAa* Δ*pucBAd/*ΔLH2A LH4	10 (0.3)	29 (5)	365 (43)

^a^ All doubling times are the averages from three independent experiments and are shown in hours (standard deviation).

## DISCUSSION

Our results suggest that flexibility in peripheral light harvesting is important for the success of *R. palustris* and related bacteria in the terrestrial soil and water habitats in which they thrive. The 20 strains analyzed here have between three and eight sets of genes to accomplish light harvesting, and most of these were expressed under the two conditions, high light and low light, that we tested. Given the high sequence identities of these small gene sets, one can speculate that gene duplication events were an important driver for diversification of light-harvesting genes. Other species of PNSB have much less diversity of LH gene inventory. One species, *Rhodospirillum rubrum*, has no peripheral LH gene sets, and another, *Rhodobacter sphaeroides*, has just two.

It is also apparent from this work that some functional redundancy exists between the LH complexes under some light intensities. However, our observation that closely related strains of a distinct *Rhodopseudomonas* clade have maintained multiple operons encoding LH complexes also suggests that each operon must be important for the fitness of the *Rhodopseudomonas* strains under some commonly encountered conditions. More work is needed to understand why the LH gene transcripts are so high under both HL and LL intensities, how the LH peptides assemble into a heterogeneous complex, and how the operons encoding the LH peptides are regulated if we are to fully understand the interplay of multiple types of LH complexes in the same organism.

## MATERIALS AND METHODS

### Bacterial strains and culture conditions.

For routine maintenance, *Rhodopseudomonas* strains were grown anaerobically in a defined mineral medium (PM) (22) supplemented with 20 mM acetate and 0.1% yeast extract in light at 30°C. Sealed culture tubes (17 ml) contained 10 ml medium, and the headspace was nitrogen gas. Cultures were initially grown anaerobically under high light intensity (30 µmol photons/m^2^/s) from a 60-W halogen light bulb (General Electric) and then diluted twice into PM medium without ammonium sulfate and supplemented with 20 mM acetate, Wolfe’s vitamins (23), and 10 µM vanadium chloride (nitrogen-fixing conditions). Vanadium chloride was added to allow growth of strains whose genes may encode only a nitrogenase that uses V as a cofactor. The cultures were then grown in the same medium under high light, low light (4 µmol photons/m^2^/s) from a 15-W halogen light bulb (General Electric), or extremely low light (<1 µmol photons/m^2^/s) from a 15-W halogen light bulb controlled by a dimmer switch. *Escherichia coli* S17-1 (24) was grown in Luria-Bertani (LB) medium at 37°C. Where appropriate, *R. palustris* was grown with gentamicin (Gm) at 100 µg/ml. *E. coli* cultures were supplemented with Gm at 20 µg/ml.

### Genome sequencing and RNA-seq analysis.

Genomic DNA was extracted using the Qiagen Genomic-tip 500/G kit and sequenced using paired-end sequencing with Illumina HiSeq 2000 (25). Sequence reads were assembled using Velvet version 1.1.07 (26). Fold coverage for sequenced genomes was 30× or greater. RNA-seq experiments and data analysis were carried out as described previously (27).

### Genetic manipulation of *R. palustris*.

In-frame deletions of *pucBAa* and *pucBAd* were created by PCR using the Phusion high-fidelity DNA polymerase (New England Biolabs) to amplify 1 kb of DNA upstream of the second codon in the coding region for each of these genes and 1 kb of DNA downstream of the stop codon for each of these genes. These fragments were then incorporated into PstI-digested (*pucBAa*) or NotI/PstI-digested (*pucBAd*) pJQ200SK suicide vector (28) using the in-Fusion PCR cloning system (Clontech). All plasmids were mobilized into *R. palustris* by conjugation with *E. coli* S17-1, and double-crossover events for deletions or allelic exchange were achieved using a previously described selection and screening strategy (29). All deletions were verified using PCR.

### Phylogenetic analysis of *pucA* and *pucB*.

Nucleotide sequences for *pucA* and *pucB* were collected from the Integrated Microbial Genomes database (https://img.jgi.doe.gov/cgi-bin/er/main.cgi). Because *pucB* homologs have a high sequence identity, they were categorized as *pucBa*, *pucBb*, *pucBc*, *pucBd*, and *pucBe* based on the *pucA* homolog contained in the same operon. The evolutionary history was inferred using the neighbor-joining method (30). The evolutionary distances were computed using the maximum composite likelihood method (31) and are in units of the number of base substitutions per site. All ambiguous positions were removed for each sequence pair. Evolutionary analyses were conducted in MEGA5 (32), and codons were aligned using MUSCLE (33). Bootstrap values are shown as percentages of 10,000 replicates. Phylogenetic trees were viewed and colored using Dendroscope (34). The amino acid alignments used to generate the phylogenetic trees are available from figshare at the following web address: http://figshare.com/articles/Marked_strain_to_strain_diversity_in_light_harvesting_antenna_complex_gene_composition_and_expression_in_photosynthetic_bacteria_affiliated_with_or_closely_related_to_Rhodopseudomonas_palustris/1597607.

### Spectrophotometric analyses.

All spectroscopy was carried out using a Beckman Coulter DU 800 spectrophotometer. Whole-cell absorption spectra of *R. palustris* were determined as described previously (3).

### Microarray data accession numbers.

The DNA sequence reads for assemblies of the genomes are available in the NCBI Short Read Archive under ID number SRP053284. Fully assembled and annotated genomes are available in the Integrated Microbial Genome database (https://img.jgi.doe.gov/cgi-bin/er/main.cgi). The Genomes Online Database (GOLD) analysis project IDs for each strain are as follows: strain 0001L, Ga0011343; strain 1a1, Ga0011382; strain AP1, Ga0011400; strain ATCC 17007, Ga0011373; strain BIS3, Ga0011374; strain CEA001, Ga0011372; strain DCP3, Ga0011383; DSM126, Ga0011344; strain DSM8283, Ga0011384; strain KD1, Ga0011330; strain NCIB8288, Ga0011345; strain RCH350, Ga0011355; strain RCH500, Ga0011356; strain RSP24, Ga0011420; strain TIE-1, Ga0030132; strain CGA009, Ga0030129; strain DX-1, Ga0030130; strain BisA53, Ga0030126; strain BisB18, Ga0030127; strain BisB5, Ga0030128. All transcriptomic data are available as raw sequencing reads deposited in NCBI Gene Expression Omnibus under the accession number GSE59544.
